# Robotic Hybrid Ablation with Novel Epicardial Electrophysiological Mapping: Impact on Atrial Fibrillation Therapy

**DOI:** 10.1093/icvts/ivaf194

**Published:** 2025-09-10

**Authors:** Zain Khalpey, Ujjawal Kumar, Mallory Taylor, Leslie Epting

**Affiliations:** Department of Cardiac Surgery, HonorHealth, Scottsdale, AZ 85258, United States; Khalpey AI Lab, Applied and Translational AI Research Institute, Scottsdale, AZ 85258, United States; Department of Cardiac Surgery, HonorHealth, Scottsdale, AZ 85258, United States; Khalpey AI Lab, Applied and Translational AI Research Institute, Scottsdale, AZ 85258, United States; School of Clinical Medicine, University of Cambridge, Cambridge, CB2 0SP, United Kingdom; Department of Electrophysiology, Abbott Inc., Chicago, IL 60064, United States; Department of Electrophysiology, Abbott Inc., Chicago, IL 60064, United States

**Keywords:** atrial fibrillation, cardiothoracic surgery, cardiovascular disorders, convergent procedure, epicardial mapping, hybrid ablation

## Abstract

We report the first use of the EnSite X system for intraoperative electrophysiological mapping during a robotic hybrid ablation (ROK-AF procedure) for long-standing persistent atrial fibrillation. Epicardial ablation targets were identified through epicardial mapping, and post-ablation electrical silencing was validated using the mapping system. Unlike conventional electrophysiological mapping systems, the orientation-independent omnipolar technology in EnSite X provides directional activation vectors, high-resolution electrograms, and peak frequency analysis, thereby enhancing substrate characterization. Post-ablation mapping confirmed the successful silencing of the posterior left atrial wall, demonstrating EnSite X’s efficacy in real-time guidance for hybrid atrial fibrillation ablation and its potential to enhance robotic surgical workflows, potentially yielding more precise ablations.

## INTRODUCTION

Atrial fibrillation (AFib) is the most common sustained arrhythmia. It is challenging to treat, especially when catheter ablations fail. Hybrid ablation offers an alternative,^1^ with our group pioneering the Robotic Convergent (ROK-AF) procedure.^2^ Precise electroanatomical mapping is critical for identifying and ablating arrhythmogenic substrates. We present the first use of the EnSite X system in a ROK-AF procedure with the Convergent Plus lesion set, comparing it to conventional systems like EnSite Precision.

## CASE REPORT

A 76-year-old male with long-standing persistent AFib despite 2 prior catheter ablations underwent a ROK-AF procedure at our institution. His medical history included hypertension, hyperlipidemia, obstructive sleep apnea, and prior percutaneous left atrial appendage occlusion. Preoperative assessments confirmed normal left ventricular function and a dilated left atrium. Preoperatively, he was on apixaban. His CHA_2_DS_2_-VASc score was 3 and his HAS-BLED and ORBIT scores were 2.

**Figure 1.**
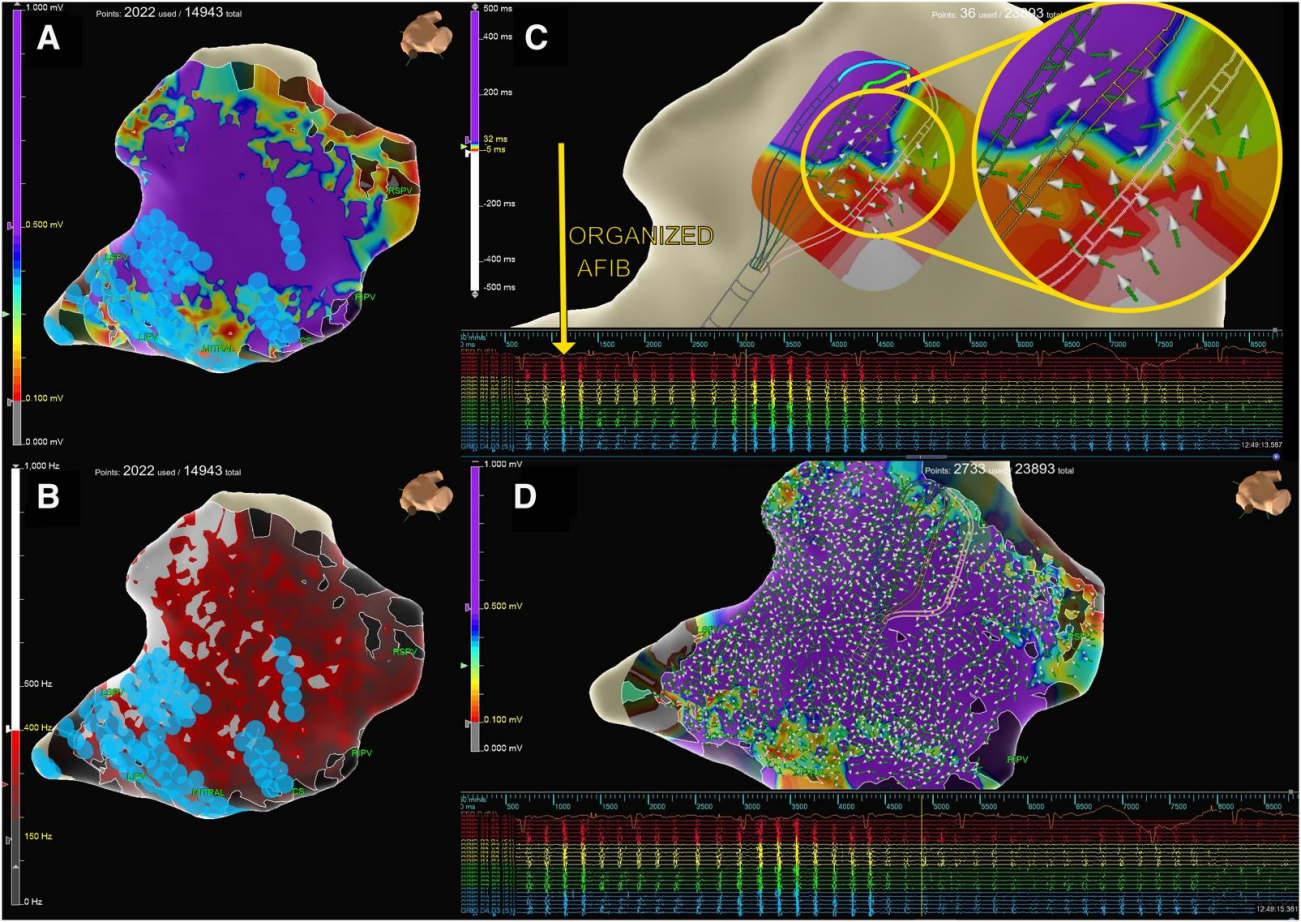
Pre-Ablation Map. (A) Voltage, (B) Peak Frequency, (C) Live View Showing AF Vector Circuits, and (D) High-Resolution 3D Activation Map

**Figure 2.**
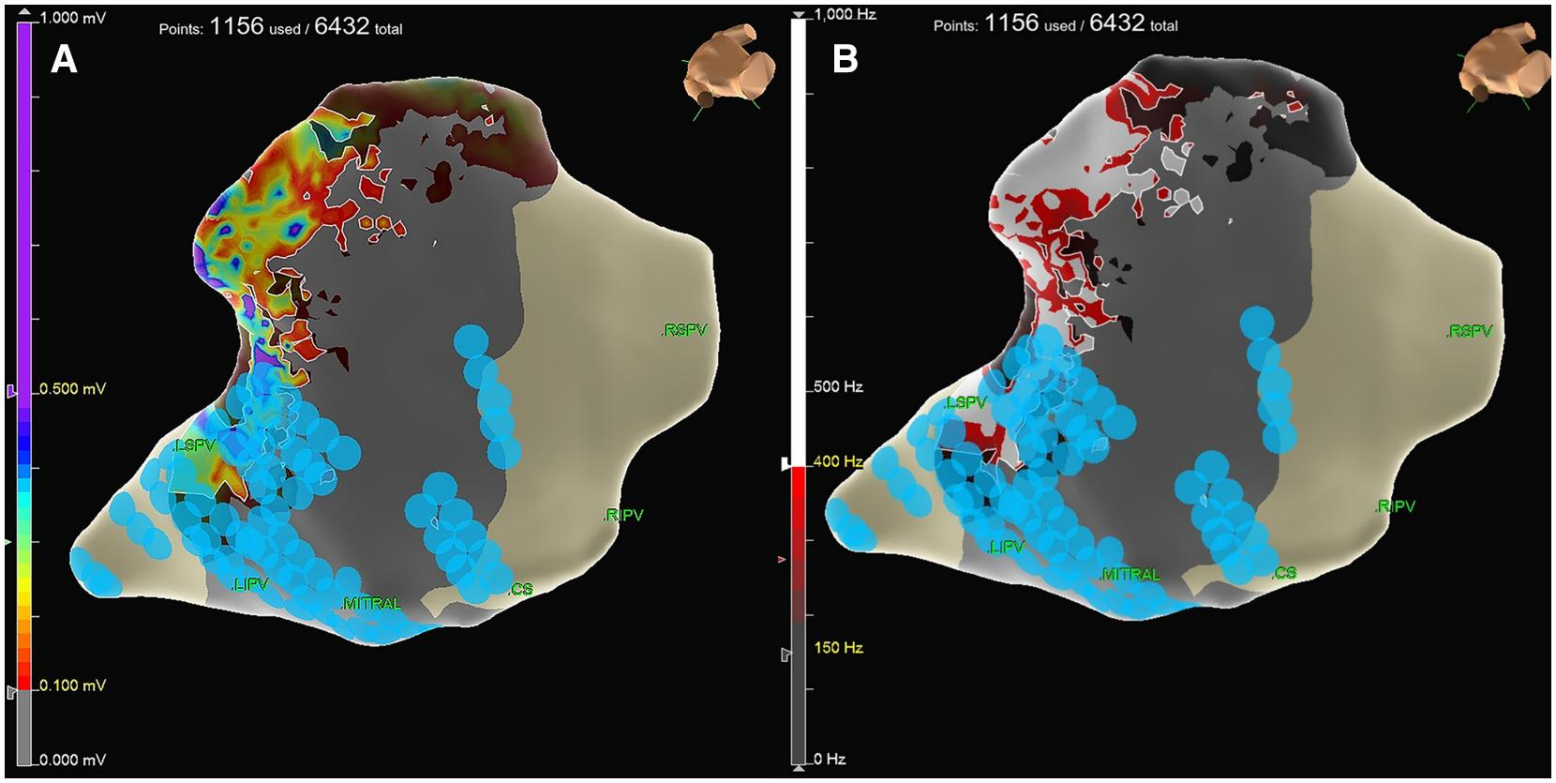
Post-Ablation Epicardial Map. (A) Voltage and (B) Peak Frequency

The procedure utilized the da Vinci Xi robotic system (Intuitive). After port placement, a mapping grid was introduced, and 3D epicardial mapping was performed using the EnSite X system (Abbott). The mapping path covered the pulmonary veins, posterior left atrial wall, generating a detailed 3D electrophysiological map (**Figure 1**). Subsequently, 23 epicardial lesions were made using the EPi-Sense ST ablation probe (AtriCure). Post-ablation mapping confirmed electrical silencing (**Figure 2**). AFib persisted on EKG, but DC cardioversion restored sinus rhythm. Pacing through the mapping tool to test and confirm the entrance and exit block confirmed the physiology and functional block for an effective lesion set. Cryoablation was performed at the intercostal spaces^3^; a chest tube was placed, and the patient was extubated in the operating room. The patient had an uncomplicated recovery, was discharged within 24 hours, and remained in sinus rhythm at 4-month follow-up.

## COMMENT

The EnSite X system, with omnipolar mapping and OT Near Field technology, offers significant advancements over EnSite Precision and traditional systems. Omnipolar mapping overcomes “bipolar blindness” by analysing signals in 360 degrees, producing comprehensive, orientation-independent maps (**Figure 1C** and **D**).^4^^,^^5^ Its peak frequency analysis (**Figure 1B**) quantifies signal sharpness, distinguishing near-field from far-field signals,^5^ improving ablation target identification where amplitude alone (**Figure 1A**) is insufficient. This is important if patients have had failed endocardial ablations in the past and can build scar maps. The live view of AF vector circuits (**Figure 1C**) enabled real-time visualization of arrhythmogenic pathways, guiding ablation strategy in real time.

EnSite X triples the resolution of EnSite Precision (36 vs 12 points per catheter position). This high-density mapping (**Figure 1D**) detailed complex substrates, enhancing ablation precision. Post-ablation maps (**Figure 2**) showed reduced voltage (**Figure 2A**) and high-frequency signals (**Figure 2B**), confirming electrical silencing of the posterior left atrial wall.

The system’s activation direction and wave speed capabilities identified slow conduction zones critical for successful ablation (**Figure 1C**). Unlike traditional systems reliant on voltage amplitude, EnSite X offers robust, reference-independent mapping, improving workflow efficiency. Rapid strategy adjustments and localized wavefront speed data minimized procedural time while maintaining high resolution.

Integration with robotic workflows enhanced substrate identification and ablation precision, reducing the risk of incomplete lesions. Compared to previous systems, EnSite X improved data collection efficiency, requiring fewer points for superior maps. However, it requires specific hardware (Advisor HD Grid Mapping Catheter) and signal quality for optimal performance, necessitating workflow adjustments.

While EnSite X demonstrated feasibility and effectiveness, its long-term outcomes require further study, with additional studies on the impact of clique technology and frequency data. While the patient remained in sinus rhythm for 4 months, the relatively short-term follow-up and single-patient nature of this study are limitations, with continued follow-up and larger patient numbers needed to show long-term success and generalizability of this technology.

## Data Availability

Patient data are confidential (cannot be shared). Anonymized data may be available upon request.
